# Multimodal single-cell network analysis uncovers BSG/CD147 as an early biomarker and signaling hub in hepatocellular carcinoma

**DOI:** 10.21203/rs.3.rs-8593693/v1

**Published:** 2026-01-27

**Authors:** Thomas DW Wang, Eun-Young K Choi, Shuo Feng, Hui Jiang, Thomas DS Wang, Sangeeta Jaiswal

**Affiliations:** University of Michigan–Ann Arbor; University of Michigan–Ann Arbor; University of Michigan–Ann Arbor; University of Michigan–Ann Arbor; University of Michigan–Ann Arbor; University of Michigan–Ann Arbor

## Abstract

**Background:**

Hepatocellular carcinoma (HCC) is a leading cause of cancer-related mortality worldwide, largely due to late-stage diagnosis and the limited sensitivity of current biomarkers such as α-fetoprotein (AFP). Early detection requires molecularly defined targets that capture the initial steps of malignant transformation. Single-cell RNA sequencing (scRNA-seq) offers high-resolution insight into tumor heterogeneity and lineage progression to enable the identification of early biomarkers. This study aimed apply scRNA-seq analysis to detect clinically important molecular patterns that define the early stages of malignant transformation in HCC and facilitate the diagnosis of small or ambiguous lesions.

**Methods:**

Two independent scRNA-seq datasets (GSE149614 and GSE189903) comprising non-tumor and HCC tissues were analyzed. Following batch correction and clustering, hepatocyte subpopulations were characterized by differential expression, pseudotime, and CytoTRACE analyses to reconstruct the trajectory from normal to malignant states. High-dimensional weighted gene co-expression network analysis (hdWGCNA) was used to identify stage-associated modules, while CellChat and protein-protein interaction analyses delineated intercellular signaling networks. Target expression was validated in paired human liver specimens using quantitative immunofluorescence.

**Results:**

scRNA-seq revealed a continuum of hepatocyte states characterized by progressive stemness and oncogenic pathway activation (MYC, E2F, G2M). Module Hep-M20 exhibited the strongest correlation with tumor stage and identified BSG/CD147 as a central hub gene with monotonic upregulation along pseudotime and strong correlation with stemness potential. CellChat analysis uncovered a cyclophilin (PPIA/PPIB)-dependent tumor-stroma signaling axis that positions BSG/CD147 as the key mediator for intercellular communication between tumor hepatocytes, fibroblasts and T cells. Ex vivo validation confirmed significantly higher BSG/CD147 protein expression in HCC versus background liver (P = 2.9×10^−11^) with excellent diagnostic accuracy (AUC = 0.93–0.96; sensitivity 86–87%; specificity 93–97%) including in lesions < 2 cm that are frequently indeterminate on conventional imaging.

**Conclusions:**

This study establishes BSG/CD147 upregulation as an early molecular event in hepatocarcinogenesis that integrates hepatocyte dedifferentiation, microenvironmental signaling, and tumor progression. Strong and specific expression in small lesions < 2 cm underscores potential as a precision biomarker and imaging target for early HCC detection, risk stratification, and therapeutic development.

## Introduction

Hepatocellular carcinoma (HCC) is a common primary liver malignancy, and a leading cause of cancer-related deaths worldwide [1, 2]. The incidence continues to rise, driven by chronic hepatitis B and C infections, metabolic liver disease, and cirrhosis [3]. Despite advances in imaging and therapy, most patients are diagnosed at an advanced stage when curative interventions, such as resection or transplantation This pathway is likely to foster a microenvironment conducive to invasion, angiogenesis, and immune modulation are no longer useful [4]. Early-stage HCC is frequently asymptomatic, and current biomarkers lack the sensitivity and specificity needed for reliable detection [5]. This limitation underscores the need for biologically based biomarkers that can identify early or indeterminate (LI-RADS 3/4) lesions on imaging to improve clinical decision-making [6].

Many imaging techniques, such as ultrasound (US), computed tomography (CT), and magnetic resonance imaging (MRI), are used in clinical practice [7] and serum biomarkers, such as α-fetoprotein (AFP) and des-γ-carboxy prothrombin (DCP), are commonly used to assist in detection and monitoring. However, diagnosing HCC at an early stage remains a significant challenge. These techniques often lack sensitivity, especially with small lesions or in the context of liver cirrhosis [8, 9]. Furthermore, false positive diagnoses may lead to unnecessary invasive procedures or undue patient anxiety. Therefore, molecular signatures, such as serum or tumor biomarkers that deliver higher sensitivity and specificity for the early and accurate detection of HCC as diagnostic tools, are greatly needed.

Tumor heterogeneity is a well-known feature of HCC, and results in high diversity at the cellular, molecular, functional, and lineage levels [10]. Cellular and molecular variability poses a significant barrier to early diagnosis [11]. This biological complexity gives rise to distinct molecular profiles even at early disease stages and in small nodules. Thus, conventional imaging phenotypes or bulk biopsy assessments may fail to capture the full molecular lesion complexity. In these contexts, molecular signature, such as gene-expression panels, pathway-activation markers, or cell-type-specific transcripts, may provide the sensitivity needed to detect small or early HCC lesions by capturing the molecular “footprint” of malignant transformation that precedes overt radiological changes [12].

Traditional bulk transcriptomic methods suffer from aggregation of signals across all cells within the sample, thereby overlooking both rare populations and interactions among distinct cell types [13]. In contrast, single-cell RNA sequencing (scRNA-seq) has revolutionized this landscape by enabling high-resolution transcriptomic analysis at the level of individual cells. This approach is capable of revealing rare hepatocyte and progenitor-like cells, reconstructing lineage trajectories, and delineating signaling networks that underlies cancer transformation. Although scRNA-seq has been used to profile immune and stromal HCC components, the full potential to identify clinically actionable early biomarkers remains unrealized. High-resolution data generated by scRNAseq allows for the discovery of candidate early biomarkers, such as specific transcripts expressed only in emerging malignant subpopulations or in the supportive microenvironmental niche, which remain undetected in bulk transcriptomic analyses. The aim of the study was to apply scRNA-seq analysis to identify clinically relevant molecular signatures that capture the process for early malignant HCC transition and support the diagnosis of small or indeterminate lesions.

## Materials and Methods

### Data acquisition from scRNA-seq datasets

Single-cell transcriptomic datasets were obtained from the Gene Expression Omnibus (GEO) repository (https://www.ncbi.nlm.nih.gov/geo) to investigate gene expression dynamics during HCC transformation. Two independent datasets were evaluated. The discovery dataset (GSE149614) consisted of liver specimens from n = 10 HCC patients distributed in 4 tissue types, including non-tumor liver, primary tumor, portal vein tumor thrombus, and metastatic lymph nodes. Only cells from non-tumor liver and primary tumor tissues were retained to focus on early cancer transformation. This cohort captured a wide range of tumor-node-metastasis (TNM) stages and etiologies, including hepatitis B virus (HBV), hepatitis C virus (HCV), and metabolic liver disease. A validation dataset (GSE189903) was used for independent module preservation analysis and included n = 4 HCC patients representing diverse tumor sizes. Single cells were prepared from different tumor regions and adjacent normal tissue. This approach enabled assessment of the robustness and reproducibility of gene co-expression network modules derived from the primary dataset across independent patient cohorts and sequencing platforms.

### Single-cell RNA-seq data processing and quality control

All analyses were conducted using R software, v4.4.0 with the Seurat v5 package [14]. Raw gene expression count matrices were imported and converted into Seurat objects. Quality control procedures were applied to exclude low-quality cells using standard filtering criteria, including the removal of cells with high mitochondrial gene content that indicate apoptotic cells, abnormally low or high gene counts that suggest empty droplets or doublets, and low total RNA expression. GSE149614 was preprocessed, and only batch correction was performed. For GSE189903, cells with nCount_RNA < 800, nFeature_RNA < 500, or mitochondrial gene percentage > 25% were excluded. These quality control steps ensured that only high-quality, biologically meaningful single-cell profiles were retained. Batch correction was performed to correct technical variations across patients and sequencing runs using the Seurat integration pipeline. Shared biological features were aligned across diverse datasets to minimize the influence of technical artifacts and ensure integrity for downstream analyses, and included clustering, differential gene expression, and pseudotime trajectory modeling.

### Cell clustering, annotation, and characterization

After initial quality filtering, the FindVariableFeatures function was used to identify highly variable genes with a focus on those that contribute most to cellular heterogeneity. The data were then normalized and centered using ScaleData, and dimensionality reduction was performed using RunPCA. The appropriate number of principal components for downstream analysis was determined with the ElbowPlot function. A shared nearest-neighbor graph was constructed using FindNeighbors. Unsupervised clustering was performed with FindClusters to identify transcriptionally distinct cell populations. For visualization in 2D space, RunTSNE and RunUMAP were applied. Cluster-specific gene expression was evaluated using FindAllMarkers for global differential expression gene (DEG) analysis across all clusters, and FindMarkers for pairwise comparisons between selected clusters. Clusters were annotated based on expression of known liver cell-type marker genes. Only clusters identified as hepatocytes were retained to focus on tumor-associated changes. Seurat visualization functions, including dot plots, violin plots, and bar plots, were used to display gene expression patterns, assess cellular composition, and compare distributions across normal and tumor samples.

### Analysis of stemness using CytoTRACE

CytoTRACE (Cellular Trajectory Reconstruction Analysis using gene Counts and Expression) was used to infer stemness and differentiation potential across normal, pro-tumor, and tumor clusters [15]. The pre-processed Seurat object from dataset GSE149614 was used for the CytoTRACE analysis. The filtered and normalized scRNA-seq data was converted into a gene expression matrix by extracting raw counts using the as.matrix() function (arkov_object[[“RNA”]]@counts). This matrix served as input to the CytoTRACE() function from the CytoTRACE R package (v0.3.3) using default parameters unless otherwise specified. CytoTRACE scores, which reflect transcriptional diversity and infer cellular plasticity, were incorporated into the Seurat object metadata. These scores were visualized on UMAP embeddings to assess differentiation gradients across clusters. Higher CytoTRACE scores corresponded to less differentiated, more stem-like states and were used to inform downstream trajectory analyses.

### Pathway enrichment analysis

Single-cell pathway enrichment analysis was performed using the escape R package (v2.2.3) on a Seurat v5 object containing hepatocytes. Enrichment scores were extracted from the “escape.ssGSEA” assay and converted into a matrix with cells in rows and pathways in columns. Average enrichment scores per pathway were computed across normal, pro-tumor, and tumor groups using dplyr. Averaged data was visualized using pheatmap by applying row-wise scaling and hierarchical clustering to highlight differential pathway activity patterns among groups.

### High-dimensional weighted gene co-expression network analysis (hdWGCNA)

hdWGCNA was performed to identify transcriptional programs associated with HCC transition. Hepatocyte subsets were first extracted from the integrated Seurat object. Using the hdWGCNA R package, a gene expression correlation matrix was computed, and a soft-thresholding power of 10 was selected to generate a scale-free weighted adjacency matrix [16]. This parameter was chosen based on the standard approach in WGCNA, where the power was selected to approximate scale-free topology, a common property of biological networks. Specifically, the scale-free topology fit index and mean connectivity across a range of powers were examined. Power = 10 was the lowest value at which networks began to exhibit scale-free behavior (R^2^ >0.85). The soft-thresholding power controls how strongly co-expression similarities (correlation values) are weighted in the adjacency matrix. Higher powers emphasize stronger correlations and suppressed weaker ones. This improved the reliability of module detection, leading to more biologically coherent gene modules. A topological overlap matrix (TOM) was then constructed to improve network robustness by emphasizing high-confidence gene-gene interactions. Gene modules were identified using average linkage hierarchical clustering and was followed by dynamic tree cutting to define module boundaries. Genes that did not cluster into any module were assigned to the gray module and indicated low or nonspecific connectivity. Module eigengenes, representative expression profiles of each module, were correlated with key clinical features, including tissue origin (normal versus tumor), tumor stage, and viral infection status (HBV/HCV) to assess potential biological relevance. Hub genes were defined as genes with the highest intramodular connectivity using the GetHubGenes function and were used to identify key regulators within each module.

### Preservation of key gene modules

Module preservation analysis was conducted using GSE189903 to assess the reproducibility of co-expression modules identified in the primary dataset (GSE149614). The GetModulePreservation function from the hdWGCNA package was employed to compute Z-summary scores, which integrate multiple metrics of module preservation and quality, including intramodular connectivity and network density. The PlotModulePreservation function was used to visualize module preservation, and display module size against Z-summary scores. Modules with Z-summary values > 10 were considered highly preserved and indicated strong reproducibility across datasets.

### Identification of stage-associated biomarkers

A multi-step integrative analysis was performed using gene co-expression modules and DEG data to identify robust biomarkers associated with HCC progression. First, hepatocyte-specific gene co-expression modules were generated using hdWGCNA. Modules were prioritized based on a strong correlation with clinical variables, such as tumor stage and tissue origin. A set of DEGs was generated by comparing gene expressions between tumor-derived and normal hepatocytes to refine candidate genes with functional relevance. The DEGs were then intersected with the gene members of prioritized modules to identify overlapping genes that were both differentially expressed and co-expressed within tumor stage-associated modules. To identify genes associated with tumor stage, DEG analysis was performed using the Seurat package with tumor stage set as the active identity class (Idents). DEG analysis was performed using the FindAllMarkers() function to restrict the analysis to a predefined list of intersecting genes. This approach identified stage-specific marker genes by comparing expression profiles across tumor stages. Expression dynamics were visualized using violin plots to allow for identification of genes that demonstrated progressive upregulation with increasing tumor stage.

### TCGA Analysis of Stage-Associated Gene Expression

Transcriptomic and clinical data for HCC were obtained from The Cancer Genome Atlas (TCGA) using the TCGAbiolinks R package. Raw gene expression data (HTSeq counts, STAR workflow) were downloaded and processed using the GDCquery, GDCdownload, and GDCprepare functions. Gene-level expression was quantified, and BSG/CD147 expression was extracted using the corresponding Ensembl gene ID. Only primary tumor samples were retained to focus on tumor-specific expression. Samples lacking valid stage annotations or gene expression were excluded from further analysis. A Spearman’s rank correlation was performed to assess the relationship between BSG/CD147 expression and tumor progression. Additionally, boxplots, violin plots, and smoothed regression lines were generated to visualize stage-wise expression differences. Outliers were identified and excluded using the IQR method for sensitivity analyses. Due to the limited number of Stage IV samples, visualizations were focused on Stages I–III to ensure balanced group comparisons.

### Trajectory analysis of biomarker expression

Pseudotime trajectory analysis was performed using the Monocle 2 R package to model the dynamic progression of hepatocyte transformation during HCC development [17]. Hepatocyte clusters previously identified using Seurat were used to construct the trajectory. These clusters were selected to capture the continuum from early to advanced tumorigenic states. Monocle 2 was applied to order cells along a pseudotime axis and infer a developmental trajectory from transcriptomic changes across individual hepatocytes. Gene expression was projected onto the trajectory to investigate the transcriptional activation patterns of candidate biomarkers. Pseudotime expression analysis was then performed to assess the onset and progression of gene activation along the transformation continuum from normal to malignancy.

### Cell-cell communication analysis

To infer and analyze intercellular communication networks, CellChat R package, v1.6.11 was used [18]. The normalized expression matrix and metadata (cell type annotations) were used to create a CellChat object via createCellChat() function. The object was then subset to include only signaling-relevant genes using subsetData() function. Analysis used the built-in CellChatDB.human ligand-receptor interaction database. In addition, BSG/CD147-PPIA and BSG/CD147-PPIB interactions were manually incorporated to investigate their role in cell-cell signaling. These interactions were curated based on known protein-protein interaction evidence, and added to the database, including the ligand-target pair: BSG/CD147 (target); PPIA, PPIB (ligands), and signaling pathway name: BSG_PPIA_PPIB. Interactions were appended to the CellChatDB$interaction and CellChatDB$complex slots prior to data subsetting. The modified database was then used to subset the expression data with subsetData() and proceed with communication probability inference.

Communication probabilities were computed using computeCommunProb(), followed by computeCommunProbPathway() and aggregateNet(), to infer and summarize pathway-specific signaling networks. Only interactions involving cell types with sufficient representation (minimum 10 cells) were retained. Visualizations such as netVisual_circle(), netVisual_bubble(), and netAnalysis_signalingRole_network() were employed to explore outgoing and incoming signaling patterns. The contribution of each ligand-receptor pair to the pathway was quantified using netAnalysis_contribution().

### Protein-protein interaction analysis

The BSG/CD147 protein-protein interaction (PPI) network was obtained from the STRING database (https://string-db.org) using a confidence score cutoff of 0.7. The network was visualized and analyzed in Cytoscape (v3.10.3). To assess context-specific interaction dynamics, expression-weighted PPI scores were calculated for normal, protumor, and tumor groups based on the average expression levels of interacting proteins and visualized using heatmap.

### Ex vivo validation of target expression in HCC

Tissue procurement and use in this study were conducted in accordance with institutional guidelines and approved by the Institutional Review Board (IRB) of University of Michigan (Approval No: HUM00248521). Formalin-fixed, paraffin-embedded (FFPE) human liver sections were obtained from the archived tissue bank in the University of Michigan Department of Pathology. Sections (5 μm thick) were cut, mounted on Superfrost Plus glass slides (Fisher Scientific), and deparaffinized. Antigen retrieval was performed in sodium citrate buffer prior to staining. Slides were blocked with 5% goat serum for 1 hour at room temperature (RT), followed by overnight incubation at 4°C with monoclonal anti-BSG/CD147 antibody (MA529060, Invitrogen) at a 1:500 dilution. After 3X washes with phosphate-buffered saline containing Tween-20 (PBST, 3 min each), sections were incubated with a Cy5.5-conjugated secondary antibody for 1 hour at RT. Slides were then washed 3X with PBST and mounted with 1.5 μm coverslips using ProLong Gold Antifade Reagent with DAPI (8961; Cell Signaling Technologies). Fluorescence images were acquired using a confocal microscope with 20X objective under identical exposure settings for tumor and background liver. Mean fluorescence intensities were quantified by placing 3X 20×20 μm^2^ boxes entirely within liver tissues using custom MATLAB software (MathWorks, Inc), while avoiding regions of saturated signal. Adjacent sections were processed for routine pathology (H&E) and independently evaluated by an expert liver pathologist (EKC). On each slide, the pathologist delineated the tumor region and adjacent background liver, which were then used to quantify the fluorescence signal.

### Statistical analysis

Unless otherwise specified, default statistical tests were used for all R functions. DEG analysis was performed using the Wilcoxon Rank Sum test to assess statistical significance. Image quantification data were analyzed using GraphPad Prism, v10.4.1. Paired t-tests were applied where appropriate to assess differences between matched groups. Receiver operating characteristic (ROC) curve analysis was used to evaluate sensitivity and specificity. Linear regression was used to assess the relationship between tumor size and target-to-background (T/B) ratio, calculated as the fluorescence intensity of the tumor (T) divided by that of the surrounding background (B) liver. Spearman analysis was performed to determine correlation coefficients.

## Results

### Data acquisition from scRNA-seq datasets

Single-cell transcriptomic analysis was performed to identify candidate biomarkers for early HCC detection. The discovery dataset (GSE149614) consisted of liver specimens from n = 10 HCC patients. Only cells from non-tumor liver and primary tumor were retained to focus on early cancer transformation. A validation cohort (GSE189903) was used for module preservation analysis. This dataset included n = 4 HCC patients with diverse tumor stages, sizes and locations, and was used to evaluate robustness and reproducibility of gene co-expression patterns derived from the primary dataset across independent patient cohorts and technical platforms.

### Single-cell RNA-seq data processing and quality control

Initial analyses revealed substantial batch effects in both datasets with cells clustering primarily by patient origin and individual sample identifiers as shown on tSNE plots, Fig. S1**A**,**B**. Batch-driven clusters were eliminated using the Seurat integration pipeline. Post-integration, cells from different patients and samples exhibited extensive intermixing, and reflected successful alignment of biologically similar cell populations, Fig. S1**C**,**D**. This correction enabled reliable interpretation of cellular heterogeneity by ensuring that clustering and downstream analyses were driven by biological variations rather than technical differences. After stringent quality control and integration, the final datasets comprised 34,414 tumor-derived and 28,687 normal liver cells (GSE149614), and 43,656 tumor-derived and 30,614 normal liver cells (GSE189903).

### Cell clustering, annotation, and characterization Identification and Annotation of Liver Cell Types

Unsupervised clustering identified 32 distinct cell clusters that represented a range of liver cell types. Annotations based on established marker genes revealed a diverse cellular composition, including hepatocytes, Kupffer cells, macrophages, dendritic cells, T cells, NK cells, B cells, fibroblasts, and endothelial cells, and reflects liver microenvironment heterogeneity, Fig. S2**A**. Stratification by tissue origin showed differential distribution of these populations between normal and tumor with several clusters enriched in either tissue type, Fig. S2**B**. Specific cell populations were preferentially represented in either normal or tumor, Fig. S2**C**. DEG analysis revealed clear transcriptional differences between normal and tumor. Tumor hepatocytes showed upregulation of genes associated with malignancy, while normal hepatocytes retained expression of genes linked to physiological liver function, Fig. S2**D**. Clusters 3, 4, 5, 8, 9, and 26 were identified as hepatocytes based on marker gene expression.

### Hepatocyte Subpopulation Analysis

tSNE analysis was used to explore hepatocyte heterogeneity between normal and tumor. Hepatocyte clusters (red) were identified based on distinct marker profiles, Fig. 1A. Cluster composition analysis revealed differential enrichment levels. Cluster 26 was composed predominantly of normal hepatocytes (N), while clusters 4, 5, and 9 were enriched in tumor (T). Clusters 3 and 8 contained cells from both normal and tumor, designated as pro-tumor (PT), and represented transitional cellular states, Fig. 1B. CytoTRACE was used to evaluate stemness properties and was found to increase in tumor versus normal and pro-tumor clusters, Fig. 1C. Dot plot analysis highlighted elevated expression of tumor-specific markers in clusters 4, 5 and 9, and expression of normal genes in cluster 26, Fig. 1D. Clusters 3 and 8 exhibited a hybrid expression pattern by co-expressing both tumor and normal markers to support their role as intermediate states during tumor progression. Pathway enrichment analysis demonstrated significant upregulation of key cancer-related pathways within tumor clusters. Pathways such as MYC, which regulates cellular proliferation and metabolism [19]; DNA repair, which maintains genomic integrity [20]; the G2M checkpoint, which ensures proper cell cycle progression and prevents propagation of damaged DNA [21]; and E2F targets, which drive DNA synthesis and cell cycle regulation [22], were all elevated, Fig. S3.

### High-dimensional weighted gene co-expression network analysis (hdWGCNA)

A soft-threshold value of 10 was chosen to build a gene network to best reflect natural biological relationships while keeping the connections simple, Fig. S4**A**-**D**. Using this network approach, 22 groups of genes that show similar activity patterns, called modules, and were identified among hepatocytes, Fig. 2A. Genes that did not fit into any module were placed in a “gray” group and showed weak or inconsistent relationships with others. A dot plot shows how these gene modules change across hepatocyte clusters as they progress from normal to tumor cells, Fig. 2B. Hep-M18 and Hep-M20 expression increased steadily from normal hepatocytes (cluster 26) to early tumor-like (clusters 3 and 8) and finally tumor-rich clusters (4, 5, 9). When comparing modules with clinical features, several meaningful patterns emerged. One group, Hep-M20, showed the strongest link to tumor stage (ρ = 0.51) to suggest a role in cancer progression. Hep-M12 was most associated with whether the tissue came from a normal or tumor, and Hep-M6 correlated most with viral infection status (HBV or HCV), Fig. 2C.

### Preservation of key gene modules

Module preservation analysis was performed using hdWGCNA to assess robustness and reproducibility for co-expression modules across diverse conditions. GSE149614 was used as the discovery network, and GSE189903 was used to validate module preservation by applying the modulePreservation() function with 20 permutations. Zsummary.pres and Zsummary.qual statistics reflect preservation of density and connectivity, respectively, Fig. 2D. Modules with Z-summary scores > 10 were considered highly preserved. Hep-M2 and Hep-M20 both met this threshold, and confirmed robustness and biological relevance across independent patient cohorts. However, the Hep-M2 module did not exhibit a clear progression from normal to tumor, and was not used to identify stage-associated biomarkers.

### Identification of stage-associated biomarkers

DEG analysis between normal and tumor hepatocytes were intersected with those from modules Hep-M20 to identify candidate biomarkers linked to HCC progression. This integrative analysis yielded 205 overlapping genes within Hep-M20, and represented a refined set of candidates likely involved in hepatocyte transformation, Fig. 3A. Stage specific DEG analysis was performed to identify the genes involved in tumor progression. Violin plots of the top 18 DEGs demonstrated stage-dependent upregulation with increased expression from normal liver to advanced tumor stages, Fig. 3B. Among these, Basigin (BSG)/CD147 emerged as a promising candidate, and its location on the cell surface makes it an accessible and practical target for molecular imaging. Target expression was significantly elevated in tumor hepatocytes versus normal liver cells, Fig. 3C. A progressive increase across tumor stages reached peak levels in advanced disease (stage IIIB-IV), Fig. 3D. TCGA data was analyzed to validate stage-associated BSG/CD147 expression. A progressive increase was observed with advanced pathology, Fig. 3E. A significant positive correlation was found between BSG/CD147 expression and tumor stage in TCGA-LIHC primary tumor samples.

### Trajectory analysis of biomarker expression

A single cell pseudotime trajectory was constructed using Monocle 2 to model transcriptional dynamics during HCC progression. The trajectory began with cluster 3 (pro-tumor hepatocytes) at the root, and extended through clusters 4, 5, and 9 to represent progressively advanced tumor states, thereby forming a continuum from early to late tumorigenesis, Fig. 4A. The arrangement of cells along pseudotime reflect their inferred developmental order with early-stage cells positioned near the root and advanced tumor cells occupying the terminal branches, Fig. 4B. Mapping the tissue origin of cells onto the trajectory further supports biological relevance. Normal cells were located primarily at the early end of the pseudotime axis, while tumor-derived cells clustered at later stages to support a temporal transition from normal to malignant hepatocytes, Fig. 4C. Gene trajectory plots demonstrated that BSG/CD147 expression increased progressively along the trajectory, Fig. 4D. This trend was further supported by smoothed scatter plots that revealed sustained and gradual gene upregulation over pseudotime, Fig. 4E. CytoTRACE scores projected onto the pseudotime trajectory revealed a gradual increase with lower scores observed at early stages and higher scores toward the terminal branches, Fig. 4F. A significant positive correlation was observed between BSG/CD147 expression and stemness potential (Spearman’s coefficient r = 0.48, P < 2.2×10^− 16^ to suggest that higher expression is associated with increased stemness, Fig. 4G.

### Cell-cell communication analysis

CellChat analysis was used to explore how different cell types communicate with each other. Key signaling pathways were identified using a database of known ligand–receptor pairs, and the strength of communication among cell groups was measured. Cell–cell communication patterns were visualized using CellChat, with interaction networks quantified by both interaction count, S5**A** and interaction weight, S5**B**. In the network map, thicker connecting lines represent more frequent or stronger interactions among cell types, Fig. S5**A**,**B**. Fibroblasts showed the highest level of communication activity, interacting with tumor cells (59 interactions, weight = 0.66), normal liver cells (53 interactions, weight = 0.70), and pro-tumor cells (40 interactions, weight = 0.41) to suggest a central role in shaping the tumor environment. A heatmap further illustrates the strength of signaling among different cell types, Fig. S5**C**. Overall, fibroblasts sent strong signals to immune cells, normal hepatocytes, and tumor cells to highlight their major role as communication “hubs” within the tissue.

**Figure 5:**
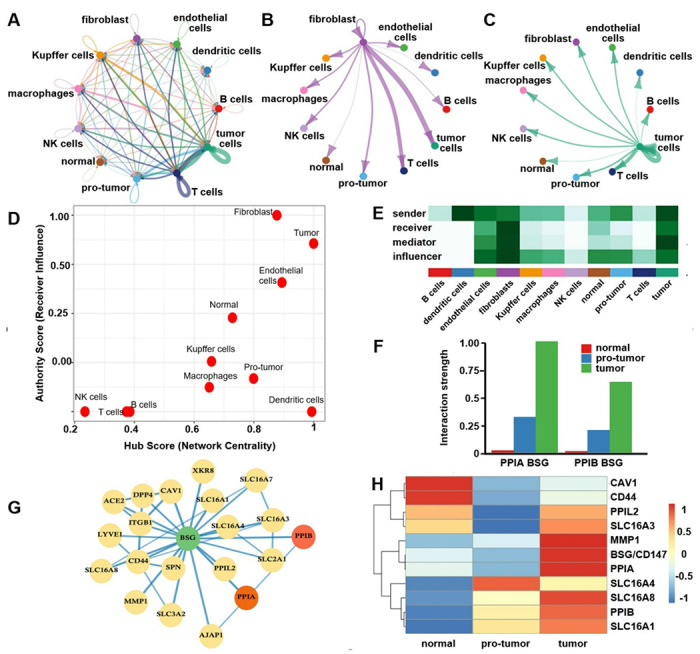
BSG/CD147 mediated cell-cell communication in tumor microenvironment. **A**) NetVisual plot of BSG/CD147 signaling illustrates communication networks among cell types. Node size corresponds to cell group abundance. Edge thickness represents relative signaling probability. **B**) Circle plot depicts the strength of fibroblast-derived signals to target cells that highlight significant communications with T and tumor cells. **C**) Circle plot shows the strongest outgoing signaling from tumor cells toward T cells and indicates a dominant tumor-to-immune cell communication axis via BSG. **D**) Authority versus hub scores reveal distinct cellular roles in BSG/CD147 signaling. Each point represents a cell population positioned according to its hub score (outgoing signaling strength) and authority score (incoming signaling strength). **E**) Signaling role heatmap depicts cellular functions within the BSG/CD147 signaling network and displays the relative sending (hub) and receiving (authority) roles of each cell population. Tumor cells show strong hub activity to indicate a central role in initiating signaling, while fibroblasts exhibit dominant receiver activity. **F**) Bar plot compares interaction strengths across normal, pro-tumor, and tumor conditions to demonstrate that tumor cells exhibit the highest signaling probability, followed by pro-tumor and normal cells. **G**) Protein-protein interaction network was derived from STRING (confidence ≥0.7) and visualized in Cytoscape. Nodes represent proteins, and edges display predicted or validated interactions. **H**) Heatmap showing the expression-weighted protein-protein interaction (PPI) scores for BSG/CD147 across normal, pro-tumor, and tumor cells. Tumor cells exhibit the highest PPI activity, followed by pro-tumor and normal cells, to indicate enhanced BSG/CD147-mediated signaling during tumor progression.

Analysis of cell-to-cell communications showed that the BSG/CD147 signaling pathway plays a major role in shaping the tumor microenvironment, Fig. 5A. Among all cell types, dendritic cells sent out signals with greatest strength (0.71), and were identified as the main “messenger” cells, while fibroblasts received the most signals (1.43) to become key “receivers” within the network, Fig. S6**A**,**B**. Strong communications was also observed from fibroblasts to both T cells and tumor cells, Fig. 5B. Tumor cells showed the highest overall signaling strength to suggest a dominant tumor-to-T cell communication pathway mediated by BSG/CD147 and cyclophilins, Fig. 5C. Tumor cells had the highest network “hub” score to highlight their central role, while fibroblasts ranked highest as signal receivers, Fig. 5D. Overall, the analysis revealed distinct cellular roles as tumor cells function as communication hubs, fibroblasts as primary recipients, and immune and endothelial cells as important regulators within the BSG/CD147-cyclophilin signaling network, Fig. 5E. The strength of cyclophilin A and B signaling (PPIA/PPIB) was further quantified across normal, pre-tumor, and tumor conditions, Fig. 5F.

### Protein-protein interaction analysis

BSG/CD147 was found to interact with several key proteins, including PPIA, PPIB, MMP1, and SLC16A1, to support its known role in matrix degradation and metabolic adaptation, Fig. 5G. Network analysis identified PPIA and PPIB as hub interactors. Expression-weighted PPI scores revealed that tumor cells displayed the highest interaction strength, followed by pro-tumor and normal groups, to reflect a progressive activation of BSG/CD147-associated signaling during tumor development, Fig. 5H.

### Ex vivo validation of target expression in HCC

BSG/CD147 expression in HCC was further validated by immunofluorescence staining of paired human liver specimens. Tumor regions exhibited markedly stronger signal intensity compared with adjacent cirrhosis or background liver, Fig. 6A,B. ROC analysis demonstrated excellent diagnostic accuracy, AUC = 0.96 with 86% sensitivity and 97% specificity, to distinguish HCC from background cirrhotic liver, Fig. 6C. A weak positive correlation was observed between tumor size and protein expression, Fig. 6D. Signal was further analyzed in HCC tumors < 2 cm in diameter to assess its potential utility for early detection, Fig. 6E. Despite the small size, these lesions showed clearly elevated fluorescence relative to background liver, Fig. 6E. ROC analysis for nodules < 2 cm demonstrated high diagnostic accuracy, AUC = 0.95 with 87.5% specificity and 93% sensitivity, Fig. 6F. Although the mean T/B ratio was higher in larger tumors, the ability of BSG/CD147 to generate detectable contrast in < 2 cm nodules underscores promise to identify early or borderline lesions that are otherwise difficult to classify radiologically (LI-RADS 3/4). Target expression also exhibited inter-patient variability, Fig. 6F, with the coefficient of variation (CV) of T/B ratios greater in large tumors (77.7%) than in small ones (40.2%) suggesting biological heterogeneity increases with tumor size, Fig. 6E. Importantly, BSG/CD147 signal remained clearly detectable within small HCC foci embedded in cirrhotic tissue to support potential utility for distinguishing indeterminate liver nodules (< 2 cm). In a representative specimen, strong fluorescence was observed in HCC tumor regions with only weak background signal in adjacent cirrhosis, Fig. S7**A**-**D**. A few isolated tumor cells within cirrhotic areas also demonstrated focal strong CD147 expression.

## Discussion

This study integrated single-cell transcriptomics, network analysis, and ex vivo validation, and identified Basigin (BSG/CD147) as a robust early HCC biomarker and signaling hub. Using two independent scRNA-seq datasets, the transcriptional trajectory was reconstructed from normal hepatocytes through pro-tumor intermediates to malignant states, and a continuum of increasing stemness and oncogenic activation was identified. Multiple gene modules associated with tumor progression were found using hdWGCNA. The Hep-M20 module showed the strongest correlation with pathological stage. BSG/CD147 emerged from within this module as a key hub gene and exhibited progressive upregulation along pseudotime. Strong correlation with stemness potential implicates gene activation as an early molecular hallmark of hepatocarcinogenesis.

Mechanistically, BSG/CD147 appears to function not only as a marker but also as a driver of early malignant transformation. Expression correlated with CytoTRACE-derived stemness to suggest that CD147 upregulation accompanies hepatocyte dedifferentiation toward progenitor-like phenotypes known to promote tumor initiation and recurrence [23]. Previous studies have shown that CD147 regulates metabolic reprogramming, extracellular matrix remodeling, and epithelial-mesenchymal transition through interactions with cyclophilins (PPIA/PPIB) and downstream MAPK/ERK, PI3K/AKT, and HIF-1α signaling cascades [24–26]. Our single-cell and network analyses extended these observations by revealing that CD147-based signaling networks mediate cross-talk between tumor hepatocytes and fibroblasts, the 2 dominant cell types of the tumor microenvironment to form a cyclophilin-dependent tumor-stroma communication axis. Additionally, our findings suggest that BSG/CD147 can engage T cells through PPIA and PPIB ligands to establish an immunomodulatory communication channel that shapes the tumor immune microenvironment. Our findings are consistent with the recent report that elevated CD147 expression in tumor cells is associated with an immunosuppressive tumor-immune microenvironment (TIME) in HCC as evidenced by increased infiltration of regulatory T cells [27]. This pathway likely fosters a microenvironment conducive to invasion, angiogenesis, and immune modulation, thereby integrating intrinsic tumor signaling with extrinsic stromal remodeling [28].

Ex vivo validation further demonstrated that BSG/CD147 expression was significantly elevated in HCC compared with adjacent background liver, including in lesions < 2 cm that are often radiologically indeterminate (LI-RADS 3/4). Our findings are consistent with the observations of a significantly elevated expression of CD147 in HCC compared to non-tumor [29]. ROC analyses confirmed high diagnostic accuracy for small tumors to underscore the potential of BSG/CD147 for early detection. Strong membrane localization, high T/B ratio, and minimal expression in surrounding cirrhosis or background liver establishes a favorable foundation for development of targeted contrast agents. Incorporating BSG/CD147 assessment into HCC surveillance frameworks could substantially enhance performance for early diagnosis and enable biological risk stratification of indeterminate nodules. Unlike prior studies that largely evaluated CD147 expression in bulk tumor tissue or serum as a static diagnostic or prognostic marker [30–32], this work uses single-cell transcriptomics and pseudotime network modelling to demonstrate CD147 upregulation as an early event in hepatocyte transformation that is correlated with increasing stemness and malignant progression.

Despite the strengths of this integrated approach, several limitations should be noted. The sample size (n = 16) for small (< 2 cm) lesions was modest, which may limit generalizability. In addition, the scRNA-seq datasets analyzed were annotated by pathological stage rather than derived directly from indeterminate nodules to restrict temporal resolution for early transformation events. Finally, while computational modeling identified plausible ligand-receptor and pathway interactions, further functional validation is needed to confirm the biological significance of BSG/CD147-mediated signaling in the tumor microenvironment.

Our results define BSG/CD147 upregulation as an early molecular event in HCC transformation to connect stemness, intercellular signaling, and microenvironmental remodeling in a unified model for early hepatocarcinogenesis. Consistent expression in small, radiologically ambiguous lesions (< 2 cm) highlights the translational potential as a precision biomarker for early HCC detection and image-guided diagnosis. Integration of BSG/CD147-targeted strategies into molecular imaging or surveillance workflows could transform the early HCC management by enabling detection, risk stratification, and intervention at the most curable stages of liver cancer.

## Data Availability

The single-cell RNA sequencing datasets analyzed during this study are publicly available from the Gene Expression Omnibus (GEO) under accession numbers GSE149614 and GSE189903. Processed data, analysis scripts, and figure source files generated during this study are available from the corresponding author upon reasonable request.

## Abbreviations

AFP: α—fetoprotein
AUC: area under the curve
BSG: Basigin
CD147: Cluster of Differentiation 147
CT: computed tomography
CV: coefficient of variation
CytoTRACE: Cellular Trajectory Reconstruction Analysis using gene Counts and Expression
DAPI: 4′,6—diamidino—2—phenylindole
DEG: differential gene expression
FFPE: formalin—fixed paraffin—embedded
GEO: Gene Expression Omnibus
GSEA: gene set enrichment analysis
H&E: hematoxylin and eosin
HBV: hepatitis B virus
HCC: hepatocellular carcinoma
HCV: hepatitis C virus
hdWGCNA: high–dimensional weighted gene co–expression network analysis
HIF: 1α–hypoxia–inducible factor 1 alpha
LIHC: Liver Hepatocellular Carcinoma (TCGA cohort)
LI: RADS–Liver Imaging Reporting and Data System
MAPK/ERK: mitogen–activated protein kinase/extracellular signal–regulated kinase
MRI: magnetic resonance imaging
PBST: phosphate–buffered saline with Tween–20
PPI: protein–protein interaction
PPIA: peptidylprolyl isomerase A (cyclophilin A)
PPIB: peptidylprolyl isomerase B (cyclophilin B)
ROC: receiver operating characteristic
RT: room temperature
scRNA: seq–single–cell RNA sequencing
TCGA: The Cancer Genome Atlas
T/B: target–to–background
TGF: β–transforming growth factor beta
tSNE: t–distributed stochastic neighbor embedding
UMAP: uniform manifold approximation and projection
US: ultrasound
WGCNA: weighted gene co–expression network analysis
